# Low levels of frailty in HIV-positive older adults on antiretroviral therapy in northern Tanzania

**DOI:** 10.1007/s13365-020-00915-3

**Published:** 2021-01-11

**Authors:** Clare Bristow, Grace George, Grace Hillsmith, Emma Rainey, Sarah Urasa, Sengua Koipapi, Aloyce Kisoli, Japhet Boni, Grace Anderson Saria, Sherika Ranasinghe, Marcella Joseph, William K. Gray, Marieke Dekker, Richard W. Walker, Catherine L. Dotchin, Elizabeta Mukaetova-Ladinska, William Howlett, Philip Makupa, Stella-Maria Paddick

**Affiliations:** 1 Faculty of Medical Sciences, Newcastle University, Newcastle upon Tyne, NE2 4HH UK; 2 Kilimanjaro Christian Medical University College, Moshi, Kilimanjaro Tanzania; 3 Mawenzi Regional Referral Hospital, Moshi, Kilimanjaro Tanzania; 4 The London School of Hygiene & Tropical Medicine, London, UK; 5 Northumbria Healthcare NHS Foundation Trust, North Tyneside General Hospital, North Shields, UK; 6 Department of Neuroscience, Psychology and Behaviour, University of Leicester, Leicester, UK; 7 Leicestershire Partnership NHS Trust, Leicester, UK

**Keywords:** HIV, Older adults, Sub-Saharan Africa, Frailty, Prevalence, Incidence

## Abstract

There are over 3 million people in sub-Saharan Africa (SSA) aged 50 and over living with HIV. HIV and combined antiretroviral therapy (cART) exposure may accelerate the ageing in this population, and thus increase the prevalence of premature frailty. There is a paucity of data on the prevalence of frailty in an older HIV + population in SSA and screening and diagnostic tools to identify frailty in SSA. Patients aged ≥ 50 were recruited from a free Government HIV clinic in Tanzania. Frailty assessments were completed, using 3 diagnostic and screening tools: the Fried frailty phenotype (FFP), Clinical Frailty Scale (CFS) and Brief Frailty Instrument for Tanzania (B-FIT 2). The 145 patients recruited had a mean CD4 + of 494.84 cells/µL, 99.3% were receiving cART and 72.6% were virally suppressed. The prevalence of frailty by FFP was 2.758%. FFP frailty was significantly associated with female gender (*p* = 0.006), marital status (*p* = 0.007) and age (*p* = 0.038). Weight loss was the most common FFP domain failure. The prevalence of frailty using the B-FIT 2 and the CFS was 0.68%. The B-FIT 2 correlated with BMI (*r* = − 0.467, *p* = 0.0001) and CD4 count in females (*r* = − 0.244, *p* = 0.02). There is an absence of frailty in this population, as compared to other clinical studies. This may be due to the high standard of HIV care at this Government clinic. Undernutrition may be an important contributor to frailty. It is unclear which tool is most accurate for detecting the prevalence of frailty in this setting as levels of correlation are low.

## Introduction

The global scale-up of combination anti-retroviral therapy (cART) has converted HIV infection from a terminal illness to a chronic disease with near-normal life expectancy. Consequently, people living with HIV (PLWH) are now an ageing population. HIV infection appears to be associated with accelerated biological ageing, beyond that of HIV-negative individuals (Appay and Rowland-Jones 2002; Arnsten et al 2007; Triant et al. 2007) leading to the premature expression of geriatric syndromes, such as frailty and sarcopenia (Desquilbet et al. 2007, 2009; Erlandson et al. 2013b; Piggott et al. 2013; Willig et al. 2016), for reasons that are not well understood. Plausible mechanisms include chronic inflammation and immune dysregulation (Desquilbet et al. 2009; Erlandson et al. 2013a; Franceschi et al. 2000; Klatt et al. 2013; Leng and Margolick. 2015) seen in normal ageing, frailty and HIV infection despite cART and suppression of viral load.

Frailty is a clinical syndrome of physiological vulnerability and inability to cope with internal or external stressors (Clegg et al. 2013) which independently predicts multiple adverse outcomes, such as falls, disability and death (Erlandson et al. 2012; Fried et al. 2001). The syndrome overlaps with, but is distinct from, comorbidity, disability and physiological ageing (Yarnall et al. 2017).

Geriatric syndromes in the ageing HIV population are a global public health problem that will soon disproportionally affect sub-Saharan Africa (SSA), due to its high burden of HIV disease and rapidly ageing population of PLWH (Pillay and Maharaj 2013). An estimated 3 million PLWH in SSA were aged 50 and over in 2011 (Negin and Cumming 2010), and this is likely to increase to 9.1 million by 2040 (Hontelez et al. 2012).

Current data on frailty in HIV in SSA are limited. No studies have focussed on older adults, and prevalence estimates vary (3.4–19.4%), with differing frailty definitions and inclusion criteria (Cournil et al. 2014; Pathai et al. 2013). We therefore have limited understanding of frailty in this newly emergent, ageing population of PLWH receiving long-term cART in SSA.

Geriatric care in SSA is greatly under-resourced (Dotchin et al. 2012). It is recommended that screening for comorbidities and complications such as frailty is undertaken alongside routine HIV follow-up (Van Damme et al. 2008). To achieve this, screening and diagnostic tools for geriatric syndromes should be culturally appropriate and be suitable for use by non-specialists in a low resource setting.

The Fried frailty phenotype (FFP) (Fried et al. 2001) is a non-specialist frailty assessment method and the most widely used phenotypic measure. The FFP has previously been used to estimate frailty in community-dwelling non-HIV older adults in rural Tanzania (Lewis et al. 2018) and examines 5 domains: weakness, slow walking speed, unintentional weight loss, low physical activity and exhaustion. Cross-cultural adaptation and modification was required in this previous Tanzanian study to ensure questions were understood, including quantification of exercise and weight loss (Lewis et al. 2018).

Another simple phenotypic measure is the Clinical Frailty Scale (CFS), a pictograph with a short clinical description widely used in high-income countries (HICs) for identification of frailty in older adults (Rockwood et al. 2005). There is no published research regarding its use in SSA.

Additionally, our team has previously developed a culturally appropriate frailty screening tool for use in non-HIV rural community-dwelling elders in Tanzania (the Brief Frailty Instrument for Tanzania (B-FIT)) (Gray et al. 2017). The B-FIT 2 was developed from a longitudinal cohort study in Northeastern Tanzania of community-dwelling people aged 70 and over, and modelling was used to identify Tanzania-specific frailty markers (Lewis et al. 2019). In that rural setting and cultural environment of subsistence farming and multigenerational living, frailty appeared to present less by physical parameters like walking speed and weight loss than in HIC studies and more by uncorrected sensory impairment, social isolation and nutritional issues (Lewis et al. 2019).

We aimed to estimate the prevalence of frailty using these three simple non-specialist measures (CFS, FFP and B-FIT 2) in a clinic-based HIV-positive cohort aged 50 and over receiving cART according to the 2017 Tanzanian HIV guidelines. We also aimed to identify important contributors and associations with frailty in this setting.

## Methods

### Ethical consideration

The study was approved nationally by the Tanzanian National Institute for Medical Research and locally by the Kilimanjaro Christian Medical College Research Ethics Committee. All potential participants were given written and verbal information about the study by study nurses trained in informed consent procedures. Procedures were agreed with the hospital administration for onward referral and handover of individuals with health issues requiring treatment identified through the course of the study.

### Participants and setting

Tanzania is a low-income country in East Africa (The World Bank 2019). The current life expectancy is 66.31 years (The World Bank 2019). The current national HIV prevalence rate is 3.9% in adults, of whom 66% are estimated to receive cART (UNAIDS 2017. Tanzanian HIV treatment guidelines were updated in 2017 and state that all HIV-positive individuals should receive cART, regardless of CD4 count (The United Republic of Tanzania 2017)*.*

This study took place in Mawenzi Regional Referral Hospital (MRRH) HIV Care and Treatment Centre (CTC) in Kilimanjaro region, Tanzania. MRRH is a government referral hospital, provides free of charge HIV treatment and has implemented the 2017 guidelines. Kilimanjaro was a ‘pioneer site’ for cART, and treatment has been available for over 15 years resulting in a stable, long-term treated population of PLWH. Kilimanjaro has a high proportion of rural subsistence farmers with a smaller proportion engaged in commercial agriculture. Levels of education are low in rural elders with a high proportion of illiteracy in women aged 70 and over and levels of self-reported disability are low with most working into old age (Dewhurst et al. 2012; Paddick et al. 2014).

### Study participants

Individuals aged 50 and over attending the CTC for routine follow-up were systematically sampled in order of arrival for routine appointments over a 10-week period (March–May 2019). Every third patient was approached for inclusion. Exclusion criteria were refusal to take part or if participation might delay necessary treatment or investigations in the opinion of the study doctor.

### Demographic and HIV disease data

Sociodemographic information (age, educational and occupational background, marital status, living arrangements) was obtained by self-report. Comorbidities and medication use were identified through self-report and crosschecked against clinic records (record of previous tuberculosis, diabetes or hypertension treatment). Blood pressure was measured during clinical assessment and hypertension defined as ≥ 140/90 mmHg based on the mean of three readings at rest (in those not previously prescribed antihypertensives). HIV disease specific data (WHO disease stage, medication regimen, nadir CD4 and date of diagnosis) were obtained through case note review. Height, weight and body mass index (BMI) as well as current HIV status with CD4 count and HIV viral load were measured at the time of recruitment. Performance status was measured using the Karnofsky performance status scale (O'Dell et al. 1995).

### Frailty assessment

#### FFP

The FFP was determined based on previous experience and adaptation of this measure in rural Tanzania (Lewis et al. 2018). The five key elements (weakness, slow walking speed, unintentional weight loss, low physical activity and exhaustion) were defined as follows:

## Weakness

Hand-grip strength (HGS) was measured in the dominant hand using a JAMAR Hydraulic Hand Dynamometer. The highest of three attempts was recorded. The weakness parameter was met if HGS was < 21 kg in males and < 10 kg in females; below the 25th centile in an African population aged 50–70 (Leong et al. 2016).

## Walking speed

Walking speed was the time taken to walk a 6-m distance on even ground. Walking aids were allowed. The 6 m was measured in the middle section of a 10-m walk and repeated 3 times to calculate an average. In a previous study in 2016, normative data had been collected in medical outpatient population at MRRH (*n* = 85) assumed to be representative of a Tanzanian population and age and education matched, and HIV negative (Paddick et al. 2017). Walking speed more than 1 standard deviation (SD) below the mean was deemed slow.

Weight loss. Weight loss was defined as > 1 kg unintentional weight loss when comparing weight recorded at time of assessment with weight recorded at the previous clinic visit.

## Physical activity

Physical activity was recorded using the International Physical Activity Questionnaire (IPAQ) (Craig et al. 2003). A positive score on this domain was recorded if 0 days of moderate physical exercise per week were self-reported.

## Exhaustion

Exhaustion was self-reported, using 2 questions from the Centre for Epidemiological Studies of Depression (CES-D) scale (Orme et al. 1986). These two questions were ‘I felt that everything I did was an effort’ and ‘I felt that I could not get going’, translated verbatim into Swahili by the Tanzanian study doctor.

Each key element was given a score of 1 if positive and 0 if negative. This gave each patient a score out of 5. A score of 1–2 was determined to be pre-frail and a score of ≥ 3 was determined to be frail.

## B-FIT 2

The B-FIT 2 measure of frailty developed for elders in rural Tanzania combines the IDEA screen (a culturally appropriate low literacy cognitive screen developed in Tanzania and validated in other SSA settings) (Gray et al. 2014, 2016; Paddick et al. 2015), Barthel index (measure of disability previously used and adapted in Tanzania by our team) (Dewhurst et al. 2012; Lewis et al. 2018; Mahoney and Barthel 1965), alongside visual acuity, calf circumference and social isolation. Visual acuity was measured at 3 m using a Landolt broken ring ‘C’ chart, designed for low literacy populations. If both eyes had a LogMAR value of ≥ 0.5, then this was considered to be moderate to severe visual impairment (Bourne et al. 2017; Stevens et al. 2013). Calf circumference was measured with a tape measure whilst the patient was seated, knee at 90° flexion. Reduced calf circumference was defined as < 31 cm, as per the original B-FIT 2. To calculate a score for social isolation, 4 questions were amalgamated (Appendix 1). The B-FIT 2 score was calculated out of 20 for each patient (Table 1).

**Table 1:** B-FIT 2 score. The allocated scores to the different components of the B-FIT 2

B-FIT 2 score	Scoring system	Assigned B-FIT 2 score
Barthel index		
Mild/no disability	19–20	0
Moderate disability	15–18	3
Severe disability	10–14	9
IDEA cognitive screen		
‘No dementia’	10–15	0
‘Possible dementia’	8–9	1
‘Probable dementia’	≤ 7	2
Calf circumference	< 31 cm	3
Severe or extreme problems joining in community activities	3–4 in social exclusion questions	5
Disability in distance vision	≥ 0.5 LogMAR in both eyes	1

### CFS

A CFS score (1–9) was given based on the appearance and presentation of the patient in relation to the pictorial graded scale at the time of clinical assessment (Rockwood et al. 2005). The study doctor, familiarised with the scale during pre-study training, assigned this score.

### Statistical methods

Statistical analyses were supported by IBM SPSS for Windows version 24. For normally distributed data, ANOVA was used. Age was treated as non-normally distributed data, due to skew. For non-normally distributed continuous data, a Kruskal–Wallis test was performed. Spearman’s rank correlations were performed to look for correlations between the B-FIT 2, CFS and sociodemographic variables, HIV disease markers and comorbidities. Analyses by gender were undertaken as, although frailty appears more common in females, it is less strongly associated with mortality (Berges et al. 2009; Zhang et al. 2018). Significance was set at 5% and two-tailed tests used throughout. The 95% confidence intervals (CIs) for prevalence in the B-FIT 2 and CFS fell below 0 but were rounded up to 0.

## Results

**Fig. 1:**
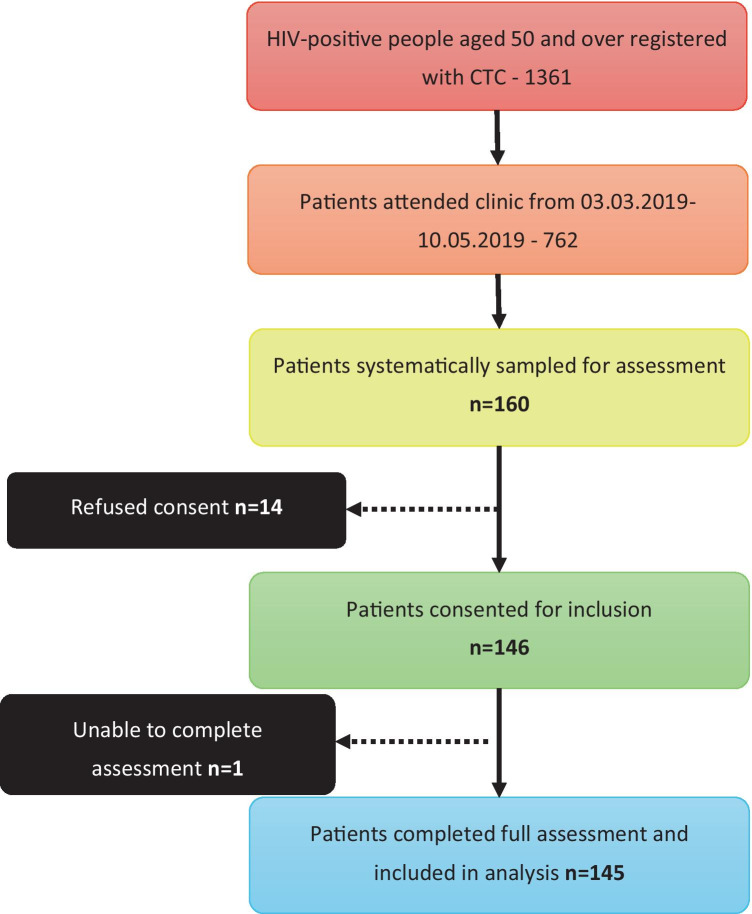
Participant selection—flowchart describing the inclusion of participants at each stage

The MRRH CTC serves 1361 HIV-positive patients aged 50 and over, of whom 982 are female (72.1%) and 379 (27.8%) male, as of March 2019. The median age is 56 (IQR 53–61). During the study period (04/03/2019 to 10/05/2019), 762 of these 1361 registered patients attended routine follow-up (526 (69.0%) female). The systematic sampling procedure selected 160 patients of whom 145 were included in the analysis with complete data (Fig. 1).

Of the 145 patients included in the sample, median age was 57 years (IQR 53–62) and 97 (66.9%) were female. Educational level was low (5.5% with no schooling, 17.9% ≤ 4 years). Only 42 (29%) were currently married, 60 (41.4%) were widowed and 35 (24.1%) separated (Table 2).

**Table 2:** Sociodemographic characteristics of the cohort

Sociodemographic characteristics	Cohort	*n*
Median age	57.0 (IQR 53.0–62.0)	145
Female gender	66.9%	97
Marital status Married Widowed Separated Never married	29.0% 41.4% 24.1% 5.5%	42 60 35 8
Living Alone	20.7%	30
Years of education 0 1–4 5–7 7 + Higher education	5.5% 12.4% 68.3% 10.3% 3.4%	8 18 99 15 5
Employment	93.8%	136
Farming employment	60.0%	87
Mean Karnofsky performance status	94.1 (SD ± 8.382)	145

### HIV disease

One hundred forty-four patients (99.3%) were on cART (one patient commenced cART on the day of assessment), of whom 77.2% (*n* = 112) were on first-line treatment. The mean CD4 count was 494.84 cells/µL, and the proportion with undetectable viral load (< 20 copies/mL) was 90 out of 124 (21 missing values). Median time since diagnosis was 10 years (IQR (interquartile range) 6–13) (Table 3).

**Table 3:** HIV characteristics of the cohort

HIV characteristics	Whole cohort	*n* =
CD4 count ± SD Missing values, *n* = 14	494.84 ± 270.40	131
Viral load suppressed Missing values, *n* = 21	72.6%	124
Median years since diagnosis (IQR)	10.00 (6.00–13.00)	145
Nadir CD4 ± SD	203.76 ± 150.74	131
World Health Organisation (WHO) HIV stage 1 2 3 4 Missing values	2.1% 16.6% 80.0% 0.7% 0.7%	3 24 116 1 1

The prevalence of visual impairment was 40.7% (defined as ≥ 0.5 in both eyes on the LogMAR scale). 1.38% (*n* = 2) had polypharmacy (defined as ≥ 4 regular medications including cART) (Rollason and Vogt, 2003). Five participants (3.45%) self-reported a previous stroke, while self-reported previous diagnoses from a healthcare professional of hypertension and/or diabetes were low at 15.86% (*n* = 23) and 4.83% (*n* = 7), respectively. The majority lived with family members (75.8%).

### Prevalence of frailty

#### FFP

Using the FFP, 4 patients (all female) met the frailty criteria, a prevalence of 2.758% (95% CI 0.093–5.425%). The prevalence of pre-frailty was 46.207% (95% CI 38.092–54.322%) (*n* = 67). Robust participants (51% (*n* = 74)) failed 0 domains. Interestingly, no participant failed more than 3 domains.

Female gender was shown to be significantly associated with frailty (*p* = 0.006), as was current marital status (being widowed, separated or never married) (*p* = 0.007) and years of education (*p* = 0.041). Age was significant associated with frailty, when treated as non-normally distributed data (*p* = 0.038) and when split into categories (*p* = 0.028) (Table 4). There was no association between frailty and HIV disease severity measures such as CD4 count, viral load, HIV stage and years since diagnosis (Table 2, Table 5).

**Table 4:** Sociodemographic and their association with frailty and pre-frailty

Characteristics	Robust		Pre-frail		Frail		Statistical test
	*n* = 74		*n* = 67		*n* = 4		
Sociodemographic							
Median age	56.0 (IQR 53.0–61.0)	74	56.0 (IQR 53–63)	67	65.0 (IQR 62.75–77.75)	4	Kruskal–Wallis test: Chi square, 6.518 *p = 0.038 *df*, 2
Age categories							Kruskal–Wallis test: Chi square, 7.166 *p = 0.028 *df*, 2
50–54 55–59 60–64 65–59 70–74 75–79 80–84	37.8% 29.7% 16.2% 8.1% 4.1% 4.1% 0.0%	28 22 12 6 3 3 0	37.3% 22.4% 20.9% 16.4% 3.0% 0.0% 0.0%	25 15 14 11 2 0 0	0.0% 0.0% 25.0% 50.0% 0.0% 0.0% 25.0%	0 0 1 2 0 0 1	
Female gender	56.8%	42	76.1%	51	100.0%	4	Mann–Whitney *U* test: *U*, 1752.0 *Z*, − 2.761 *p = 0.006
Marital status							Mann–Whitney *U* test *U*, 1634.0 *Z*, − 2.680 *p = 0.007
Married Not married	39.2% 60.8%	29 45	17.9% 82.1%	12 55	25.0% 75.0%	1 3	
Years of education							Kruskal–Wallis test: Chi square, 6.382 *p = 0.041 *df*, 2
0 1–4 5–7 7 + Higher education	8.1% 6.8% 67.8% 12.2% 4.1%	6 5 50 9 4	3.0% 14.9% 71.6% 9.0% 1.5%	2 10 48 6 1	0.0% 75.0% 25.0% 0.0% 0.0%	0 3 1 0 0	
BMI ± SD	23.70 ± 4.37	74	23.66 ± 5.532	67	19.33 ± 2.689	4	One-way ANOVA: *F* (2, 142), 1.526 *p* = 0.221

*Statistically significant (*p* ≤ 0.05) results

**Table 5:** HIV characteristics and their association with frailty and prefrailty

Characteristics	Robust		Pre-frail		Frail		Statistical test
	*n* = 74		*n* = 67		*n* = 4		
HIV factors							
CD4 count ± SD	469.95 ± 263.95	64	529.54 ± 280.552	63	364.50 ± 104.800	4	One-way ANOVA: *F* (2, 128), 1.400 *p* = 0.250
Mean years since diagnosis	9.25 ± 4.545	74	8.83 ± 4.543	67	9.75 ± 3.304	4	One-way ANOVA: *F* (2, 139), 0.343 *p* = 0.710
Viral load ± SD	6395.55 ± 33,489.42	64	13,659.70 ± 56,896.047	56	9190.0 ± 14,065.036	4	One-way ANOVA: *F* (2, 121), 0.383 *p* = 0.683
Nadir CD4 ± SD	191.95 ± 142.406	66	220.21 ± 161.293	62	123.33 ± 41.525	3	One-way ANOVA *F* (2, 121), 0.383 *p* = 0.683
WHO HIV stage							Kruskal–Wallis test: Chi square, 0.898 *p* = 0.638 *df*, 2
I II III IV Missing data	4.1% 13.5% 82.4% 0.0%	3 10 61 0	0.0% 20.9% 76.1% 1.5% 1.5%	0 14 51 1 1	0.0% 0.0% 100.0% 0.0%	0 0 4 0	

### Clinical Frailty Scale

**Fig. 2:**
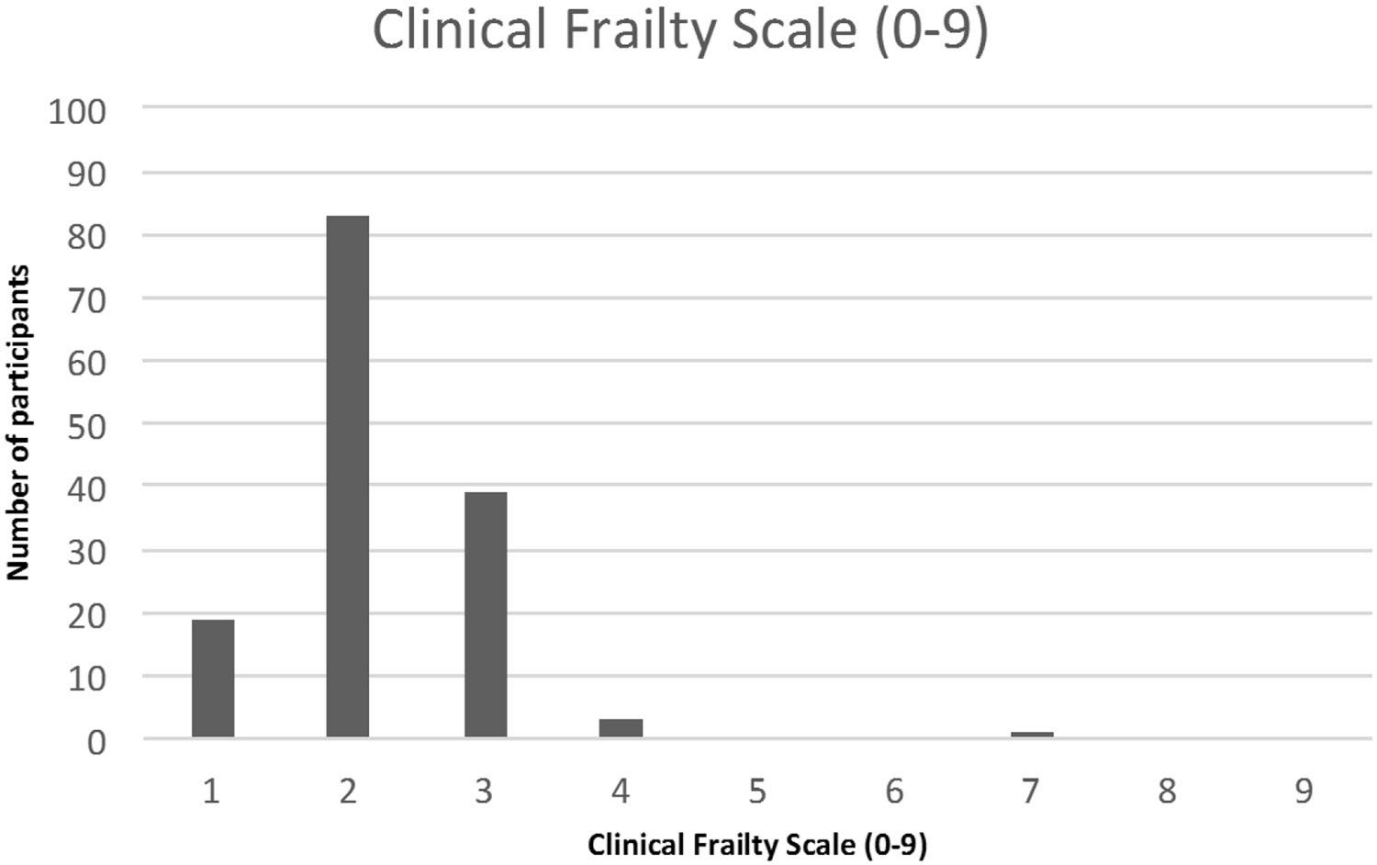
CFS scores in the cohort

Using the CFS, the prevalence of frailty (CFS score 5–9) was 0.68% (CI − 0.66–2.04%)—1 person was determined to be severely frail (CFS score 7) and 4 people vulnerable (CFS score 4) (Fig. 2). CFS score was associated with years since diagnosis *r* = 0.392 (*p* = 0.06) and age *r* = 0.349 (*p* = 0.015) in males.

#### B-FIT 2

Using the previously recommended cutoff score of 8/20, 0.68% (CI − 0.66–2.04%) (*n* = 1) met criteria for frailty. For analysis, the B-FIT 2 was treated as an ordinal scale. The median B-FIT 2 score was 1 (IQR 0–3), and the mean B-FIT 2 score was 1.48 ± 2.180. A total of 63 participants scored 0 (43.4%), and 1 participant scored 19 (0.7%). Increasing age (*r* = 0.233, *p* = 0.007) and greater years since HIV diagnosis (*r* = 0.211, *p* = 0.012) were weakly correlated with B-FIT 2 score. Age, split into 5-year categories additionally correlated with B-FIT 2 score (*r* = 2.99, *p* = 0.006). There was an inverse relationship between BMI and B-FIT 2 score (*r* = − 0.467, *p* = 0.0001) and a weak negative correlation between CD4 count and B-FIT 2 score in females (*r* = − 0.244, *p* = 0.022) (Table 6).

**Table 6:** Correlations of the B-FIT 2 with demographic and HIV-disease factors

Demographic	Correlation with B-FIT 2 score in cohort *R* = (*p* =)	Correlation with B-FIT 2 in males *R* = (*p* =)	Correlation with B-FIT 2 in females *R* = (*p* =)
Age	0.233 (0.007*)	0.271 (0.063)	0.236 (0.02*)
Age—into 5-year categories	0.299 (0.006*)	0.331 (0.031*)	0.229 (0.024*)
BMI	− 0.467 (0.0001*)	− 0.437(0.002*)	− 0.624 (0.0001*)
Educational attainment	− 0.233 (0.005*)	No correlation − 0.033 (0.825)	− 0.272 (0.007*)
HIV factors			
Years since diagnosis	0.211 (0.012*)	0.323 (0.027*)	No correlation 0.194 (0.059)
CD4 count	No correlation − 0.068(0.437)	No correlation 0.063 (0.689)	− 0.244 (0.022*)
Viral load	No correlation − 0.067 (0.467)	No correlation − 0.089 (0.565)	No correlation − 0.037 (0.758)

*Significant *p* values

## Discussion

The worldwide prevalence of frailty in HIV ranges from 3.5 to 17%, though most data originate in HIC. There have been two previous studies of frailty in HIV completed in SSA. Both studies used the FFP as a measure of frailty. A study in South Africa reported a prevalence rate of 19.4% (Pathai et al. 2013). The study cohort was comparable, with a similar median CD4 of 468 cells/μL, and 84.3% had an undetectable viral load. The median age was 42.6, lower than the median age of 57.0 in this study, although the proportion receiving cART was lower (87.1%). Another study in Senegal reported a frailty prevalence of 3.5% (Cournil et al. 2014) in a cohort with a mean age of 46.8 but details of HIV-disease severity remain unclear.

The frailty prevalence of 2.76% using the FFP in this study were lower than most worldwide studies, demonstrating a considerable lack of frailty within this population. The B-FIT 2 and CFS resulted in an even lower prevalence (0.68%) through identification of the same, single very frail individual. As the selection criteria was adults aged 50 and over, the median age was higher than other studies of frailty in HIV from SSA. Frailty was significantly associated with age using the FFP (*p* = 0.038) and the B-FIT 2 (*r* = 0.233 (*p* = 0.007)), which is unsurprising as ageing is inextricably linked with frailty. The low level of frailty in our study is supported by previous studies indicating a low rate of disability in older Tanzanians (Dewhurst et al. 2012). The population that reach older age in northern Tanzania may be relatively fit, and this may be true of PLWH. The high employment rate of 93.8% (of whom 60% are farmers, a physically demanding occupation) further supports this low level of functional impairment. The prevalence of frailty in non-HIV community-dwelling older adults in Tanzania (over the age of 60) was found to be 11.2% (Lewis et al. 2018). The prevalence of frailty in our cohort of individuals over 60 was 7.27% (95% CI 0.40952–14.13593%). Although the characteristics of these populations are not directly comparable due to the community setting of the aforementioned study, it does provide further support for the lack of frailty in this cohort. The accessibility of health services and regular review by health professionals may be protective against frailty in this well-controlled HIV population.

Rates of polypharmacy were low. Only 1.38% of the cohort was taking 4 or more medications including cART. It is unclear whether this reflects low prevalence of other comorbid conditions or lack of access to healthcare and medications. The prevalence of self-reported comorbidities (hypertension and diabetes) was low, but self-report is known to be of limited accuracy in non-communicable and chronic disease in areas of SSA where healthcare access may be limited as previously demonstrated in Tanzania (Dewhurst et al. 2013; Hunter et al. 2012).

In HIC, geriatric interventions and the advanced standard of care may allow survival with frailty and disability beyond what is possible in northern Tanzania, and extremely frail individuals may have a reduced life expectancy in Tanzania; this can also explain the low rate of frailty prevalence in the current study.

The demographic and risk factor profile in our study differs from cohorts seen in HIC. The predominant mode of HIV transmission in SSA is heterosexual sex. Therefore, the risk profile and comorbidities associated with intravenous drug use (IVDU) such as hepatitis C coinfection may not be as common in older PLWH in Tanzania. In our cohort, females made up two thirds of the participants, reflecting the demographic proportions seen in PLWH in Tanzania (29). However, the prevalence of frailty was still lower than multiple all-female cohorts, such as the Women’s Interagency HIV studies (Gustafson et al. 2016; Terzian et al. 2009). Epidemiological data suggest that frailty is more prevalent in females, and yet associated with greater mortality in males (Berges et al. 2009; Zhang et al. 2018). This was reflected in our findings, as being female was significantly associated with frailty (*p* = 0.006).

Social isolation and exclusion are important contributors to frailty (Dent and Hoogendijk. 2014; Holt-Lunstad et al. 2010; Temkin-Greener et al. 2004). In this setting, 75.8% of the cohort lived with family and may therefore be less susceptible to the isolation and loneliness that add to frailty and are experienced by many older people worldwide. In support of this is also our finding that frailty was associated with being unmarried in this cohort (*p* = 0.007).

Although chronic comorbidity was uncommon, the B-FIT 2 highlighted uncorrected sensory impairment as an important contribution to frailty. In our cohort, 40.7% had uncorrected visual impairment. This could be an area to target for frailty intervention, as the provision of glasses is relatively inexpensive and effective.

The strongest significant correlation with the B-FIT 2 was BMI (*r* = − 0.467, *p* = 0.001). As calf circumference is a part of the B-FIT 2, this is expected. However, in the FFP, the most common domain failure was weight loss, failed by 20.1%. Undernutrition and weight loss may result from limited diversity and amount of food, which is dependent on crop yield and financial stability. Food insecurity exacerbates loss of energy and diminished physiological reserves in frailty. Previous work in a related cohort suggests that malnutrition is relatively uncommon in this cohort; therefore, weight loss may also be a sign of HIV disease severity (Webb 2018). The Senegalese paper that reported a frailty prevalence of 3.5% noted the association between low body weight and frailty (Cournil et al. 2014). There was a weak correlation between CD4 count in females and frailty *r* = 0.0244 (*p* = 0.02) which may support the concept of accelerated ageing in HIV infection.

All three methods resulted in a low prevalence of frailty in this cohort supporting the notion that this older HIV + population were relatively fit. The application of the FFP in this study was modelled on a previous study which operationalised the FFP in rural community-dwelling older Tanzanians (Lewis et al. 2018) and may not be as relevant in this urban, young clinical population.

This was a well-managed clinical cohort, almost all of whom were receiving cART and of whom over two thirds had a suppressed viral load. This compares favourably to data for Tanzania as a whole. In Tanzania, the percentage of people with HIV receiving cART is 66%, with 48% achieving a suppressed viral load (UNAIDS 2017). This is likely to have contributed to the low levels of frailty seen in this study.

## Limitations

The prevalence of frailty reported here may be an underestimate. The data collection for this study took place in a clinic in Moshi, which provides HIV care for the Kilimanjaro region. Car ownership is rare in Tanzania, and as a result of this, long journeys on deteriorating roads on poor quality and overcrowded public transport are necessary to attend the clinic. In addition, stigma-related issues result in patients travelling from rural villages to Moshi to avoid having HIV status being inadvertently disclosed through being seen attending local services. Completing this journey requires a certain level of physical function, cognitive ability and robustness. We were not able to follow-up registered patients failing to attend appointments or completely lost to follow-up by undertaking a home visit, though we made several attempts to contact them by telephone. Staff reported that occasionally individuals sent relatives to collect their medication if they were too frail to do so. However, this was not routinely documented and could not be verified by clinic records. The patients lost to follow-up are likely to have been frail and/or unwell, but the cohorts assessed are likely to be typical of those attending follow-up care in this and similar settings.

Comorbidities were identified through self-report, a method known to be inaccurate in areas of limited healthcare access. We identified frailty using three different screening measures, but a more comprehensive and accurate method would have been to employ Comprehensive Geriatric Assessment (CGA), and future studies should compare the performance of these screening tools to CGA in order to determine the most accurate frailty screening tool for this setting.

Each method of measuring frailty has its limitations, which may account for the discrepancies in the frailty prevalence. The B-FIT 2 includes the IDEA screen as a measure of cognitive impairment in this population. The IDEA screen validation took place in participants over 65—higher scores were associated with younger age and education status (Gray et al. 2016) (Gray et al. 2014). Therefore, the IDEA screen may be insensitive for detecting cognitive impairment in a younger, more educated population, thus artificially lowering the prevalence of frailty. Additionally, the CFS may be culturally inappropriate, as the pictograms depict Zimmer frames and wheelchairs, which are not readily available. There is no formal social care in Tanzania. Individuals of the same frailty may not receive the same help with IADLs and ADLs as in HICs, as it is not available, lowering the CFS score.

A longitudinal study in this clinic could characterise transition from pre-frailty to frailty and assess risk factors for frailty in this setting.

## Conclusions

The prevalence of frailty in this cohort was low, reflecting the health status of this well-managed older HIV + population in Northern Tanzania. Despite there being a high burden of visual impairment, there was limited other comorbidity and disability in this cohort of older people living with HIV and attending routine follow-up care. This cohort appear to have well-managed HIV disease in a typical Tanzanian Government clinic, encouraging for future services. It may be that multigenerational living and the low prevalence of living alone is protective against isolation, which is a contributor to frailty. Weight loss is associated with frailty in this population.

## Role of the funding source

The sponsors of this study had no role in designing the study; in the collection, analysis, and interpretation of data; in the writing of the report; or in the decision to submit the paper for publication.

## Appendix 1

**Table 7:** Social isolation as a part of B-FIT 2. Questions amalgamated to create a social isolation score out of 4AQ

Question regarding social exclusion	Answer	Score
Household status	Living alone	1 point
Does the subject participate in religious and community meetings? (collateral history – see appendix 5)	No	1 point
‘They preside over feasts and ceremonies’ (instrumental ADL screen-collateral history)	– They can do this with much assistance – They cannot do this	1 point
Do you prefer to stay at home or do new things? (EURO-D)	Stay at home	1 point
Total score		/4

## References

[CR1] Appay V, Rowland-Jones SL (2002). Premature ageing of the immune system: the cause of AIDS?. Trends Immunol.

[CR2] Arnsten JH, Freeman R, Howard AA, Floris-Moore M, Lo Y, Klein RS (2007). Decreased bone mineral density and increased fracture risk in aging men with or at risk for HIV infection. Aids.

[CR3] Berges I-M, Graham JE, Ostir GV, Markides KS, Ottenbacher KJ (2009). Sex differences in mortality among older frail Mexican Americans. Journal of Women's Health.

[CR4] Bourne RRA, Flaxman SR, Braithwaite T, Cicinelli MV, Das A, Jonas JB, Keeffe J, Kempen JH, Leasher J, Limburg H, Naidoo K, Pesudovs K, Resnikoff S, Silvester A, Stevens GA, Tahhan N, Wong TY, Taylor HR, Bourne R, Ackland P, Arditi A, Barkana Y, Bozkurt B, Braithwaite T, Bron A, Budenz D, Cai F, Casson R, Chakravarthy U, Choi J, Cicinelli MV, Congdon N, Dana R, Dandona R, Dandona L, Das A, Dekaris I, Del Monte M, Deva J, Dreer L, Ellwein L, Frazier M, Frick K, Friedman D, Furtado J, Gao H, Gazzard G, George R, Gichuhi S, Gonzalez V, Hammond B, Hartnett ME, He M, Hejtmancik J, Hirai F, Huang J, Ingram A, Javitt J, Jonas J, Joslin C, Keeffe J, Kempen J, Khairallah M, Khanna R, Kim J, Lambrou G, Lansingh VC, Lanzetta P, Leasher J, Lim J, Limburg H, Mansouri K, Mathew A, Morse A, Munoz B, Musch D, Naidoo K, Nangia V, Palaiou M, Parodi MB, Pena FY, Pesudovs K, Peto T, Quigley H, Raju M, Ramulu P, Resnikoff S, Robin A, Rossetti L, Saaddine J, Sandar MYA, Serle J, Shen T, Shetty R, Sieving P, Silva JC, Silvester A, Sitorus RS, Stambolian D, Stevens G (2017). Magnitude, temporal trends, and projections of the global prevalence of blindness and distance and near vision impairment: a systematic review and meta-analysis. The Lancet Global Health.

[CR5] Clegg A, Young J, Iliffe S, Rikkert MO, Rockwood K (2013). Frailty in elderly people. The Lancet.

[CR6] Cournil A, Eymard-Duvernay S, Diouf A, groupe d’étude de la cohorte A (2014). Vieillissement osseux et syndrome de fragilité à 10 ans de traitements ARV au Sénégal. Bulletin de la Société de pathologie exotique.

[CR7] Craig CL, Marshall AL, Sjostrom M, Bauman AE, Booth ML, Ainsworth BE, Pratt M, Ekelund U, Yngve A, Sallis JF, Oja P (2003). International physical activity questionnaire: 12-country reliability and validity. Med Sci Sports Exerc.

[CR8] Dent E, Hoogendijk EO (2014). Psychosocial factors modify the association of frailty with adverse outcomes: a prospective study of hospitalised older people. BMC Geriatr.

[CR9] Desquilbet L, Jacobson LP, Fried LP, Phair JP, Jamieson BD, Holloway M, Margolick JB (2007). HIV-1 infection is associated with an earlier occurrence of a phenotype related to frailty. The Journals of Gerontology: Series A.

[CR10] Desquilbet L, Margolick JB, Fried LP, Phair JP, Jamieson BD, Holloway M, Jacobson LP, Multicenter ACS (2009). Relationship between a frailty-related phenotype and progressive deterioration of the immune system in HIV-infected men. J Acquir Immune Defic Syndr.

[CR11] Dewhurst F, Dewhurst MJ, Gray WK, Orega G, Howlett W, Chaote P, Dotchin C, Longdon AR, Paddick S-M, Walker RW (2012). The prevalence of disability in older people in Hai, Tanzania. Age Ageing.

[CR12] Dewhurst MJ, Dewhurst F, Gray WK, Chaote P, Orega GP, Walker RW (2013). The high prevalence of hypertension in rural-dwelling Tanzanian older adults and the disparity between detection, treatment and control: a rule of sixths?. J Hum Hypertens.

[CR13] Dotchin CL, Akinyemi RO, Gray WK, Walker RW (2012). Geriatric medicine: services and training in Africa. Age Ageing.

[CR14] Erlandson KM, Allshouse AA, Jankowski CM, Duong S, MaWhinney S, Kohrt WM, Campbell TB (2012). Risk factors for falls in HIV-infected persons. JAIDS Journal of Acquired Immune Deficiency Syndromes.

[CR15] Erlandson KM, Allshouse AA, Jankowski CM, Lee EJ, Rufner KM, Palmer BE, Wilson CC, MaWhinney S, Kohrt WM, Campbell TB (2013). Association of functional impairment with inflammation and immune activation in HIV type 1-infected adults receiving effective antiretroviral therapy. J Infect Dis.

[CR16] Erlandson KM, Allshouse AA, Jankowski CM, MaWhinney S, Kohrt WM, Campbell TB (2013). Functional impairment is associated with low bone and muscle mass among persons aging with HIV infection. J Acquir Immune Defic Syndr.

[CR17] Franceschi C, BonafÈ M, Valensin S, Olivieri F, De Luca M, Ottaviani E, De Benedictis G (2000). Inflamm-aging: an evolutionary perspective on immunosenescence. Ann N Y Acad Sci.

[CR18] Fried LP, Tangen CM, Walston J, Newman AB, Hirsch C, Gottdiener J, Seeman T, Tracy R, Kop WJ, Burke G, McBurnie MA (2001). Frailty in older adults: evidence for a phenotype. J Gerontol A Biol Sci Med Sci.

[CR19] Gray WK, Orega G, Kisoli A, Rogathi J, Paddick S-M, Longdon AR, Walker RW, Dewhurst F, Dewhurst M, Chaote P, Dotchin C (2017). Identifying frailty and its outcomes in older people in rural Tanzania. Exp Aging Res.

[CR20] Gray WK, Paddick S-M, Kisoli A, Dotchin CL, Longdon AR, Chaote P, Samuel M, Jusabani AM, Walker RW (2014). Development and Validation of the Identification and Intervention for Dementia in Elderly Africans (IDEA) Study Dementia Screening Instrument. J Geriatr Psychiatry Neurol.

[CR21] Gray WK, Paddick SM, Collingwood C, Kisoli A, Mbowe G, Mkenda S, Lissu C, Rogathi J, Kissima J, Walker RW, Mushi D, Chaote P, Ogunniyi A, Dotchin CL (2016). Community validation of the IDEA study cognitive screen in rural Tanzania. International Journal of Geriatric Psychiatry.

[CR22] Gustafson DR, Shi Q, Thurn M, Holman S, Minkoff H, Cohen M, Plankey MW, Havlik R, Sharma A, Gange S, Gandhi M, Milam J, Hoover D (2016). Frailty and constellations of factors in aging HIV-infected and uninfected women-The Women’s Interagency HIV Study. The Journal of frailty & aging.

[CR23] Holt-Lunstad J, Smith TB, Layton JB (2010). Social relationships and mortality risk: a meta-analytic review. PLOS Medicine.

[CR24] Hontelez JA, de Vlas SJ, Baltussen R, Newell ML, Bakker R, Tanser F, Lurie M, Barnighausen T (2012). The impact of antiretroviral treatment on the age composition of the HIV epidemic in sub-Saharan Africa. Aids.

[CR25] Hunter E, Rogathi J, Chigudu S, Jusabani A, Jackson M, McNally R, Gray W, Whittaker RG, Iqbal A, Birchall D, Aris E, Walker R (2012). Prevalence of active epilepsy in rural Tanzania: a large community-based survey in an adult population. Seizure.

[CR26] Klatt NR, Chomont N, Douek DC, Deeks SG (2013). Immune activation and HIV persistence: implications for curative approaches to HIV infection. Immunol Rev.

[CR27] Leng SX, Margolick JB (2015). Understanding frailty, aging, and inflammation in HIV infection. Current HIV/AIDS Reports.

[CR28] Leong DP, Teo KK, Rangarajan S, Kutty VR, Lanas F, Hui C, Quanyong X, Zhenzhen Q, Jinhua T, Noorhassim I, AlHabib KF, Moss SJ, Rosengren A, Akalin AA, Rahman O, Chifamba J, Orlandini A, Kumar R, Yeates K, Gupta R, Yusufali A, Dans A, Avezum Á, Lopez-Jaramillo P, Poirier P, Heidari H, Zatonska K, Iqbal R, Khatib R, Yusuf S (2016). Reference ranges of handgrip strength from 125,462 healthy adults in 21 countries: a prospective urban rural epidemiologic (PURE) study. Journal of cachexia, sarcopenia and muscle.

[CR29] Lewis EG, Coles S, Howorth K, Kissima J, Gray W, Urasa S, Walker R, Dotchin C (2018). The prevalence and characteristics of frailty by frailty phenotype in rural Tanzania. BMC Geriatr.

[CR30] Lewis EG, Whitton LA, Collin H, Urasa S, Howorth K, Walker RW, Dotchin C, Mulligan L, Shah B, Mohamed A, Mdegella D, Mkodo J, Zerd F, Gray WK (2019). A brief frailty screening tool in Tanzania: external validation and refinement of the B-FIT screen. Aging Clinical and Experimental Research. 10.1007/s40520-019-01406-0 31811571

[CR31] Mahoney FI, Barthel DW (1965). Functional evaluation: the Barthel index. Md State Med J.

[CR32] Negin J, Cumming RG (2010). HIV infection in older adults in sub-Saharan Africa: extrapolating prevalence from existing data. Bull World Health Organ.

[CR33] O'Dell MW, Lubeck DP, O'Driscoll P, Matsuno S (1995). Validity of the Karnofsky performance status in an HIV-infected sample. J Acquir Immune Defic Syndr Hum Retrovirol.

[CR34] Orme JG, Reis J, Herz EJ (1986). Factorial and discriminant validity of the Center for Epidemiological Studies Depression (CES-D) scale. J Clin Psychol.

[CR35] Paddick S-M, Gray WK, Ogunjimi L, Lwezuala B, Olakehinde O, Kisoli A, Kissima J, Mbowe G, Mkenda S, Dotchin CL, Walker RW, Mushi D, Collingwood C, Ogunniyi A (2015). Validation of the Identification and Intervention for Dementia in Elderly Africans (IDEA) cognitive screen in Nigeria and Tanzania. BMC geriatrics.

[CR36] Paddick S-M, Longdon A, Gray WK, Dotchin C, Kisoli A, Chaote P, Walker R (2014). The association between educational level and dementia in rural Tanzania. Dementia & neuropsychologia.

[CR37] Paddick SM, Kellet-Wright J, Flatt A, Eaton P, Kisoli A, Thornton J, Irwin C, McCartney J, Yarwood V, Walker R, Dotchin C, Gray WK, Lwezuala B, Mukaetova-Ladinska E, Akinyemi R, Urasa S (2017). Prevalence of HIV-associated neurocognitive impairment (hand) amongst adults aged 50 and over attending a HIV clinic in Northern Tanzania. J Neurol Sci.

[CR38] Pathai S, Gilbert C, Weiss HA, Cook C, Wood R, Bekker L-G, Lawn SD (2013). Frailty in hiv-infected adults in South Africa. JAIDS Journal of Acquired Immune Deficiency Syndromes.

[CR39] Piggott DA, Muzaale AD, Mehta SH, Brown TT, Patel KV, Leng SX, Kirk GD (2013). Frailty, HIV infection, and mortality in an aging cohort of injection drug users. PLoS ONE.

[CR40] Pillay NK, Maharaj P (2013). Population ageing in Africa. In: Aging and health in Africa. Maharaj P, (ed). Springer US: Boston, MA, pp 11–51.

[CR41] Rockwood K, Song X, MacKnight C, Bergman H, Hogan DB, McDowell I, Mitnitski A (2005). A global clinical measure of fitness and frailty in elderly people. CMAJ : Canadian Medical Association journal = journal de l'Association medicale canadienne 173: 489–495. 10.1503/cmaj.050051 PMC1188185 16129869

[CR42] Rollason V, Vogt N (2003). Reduction of polypharmacy in the elderly. Drugs Aging.

[CR43] Stevens GA, White RA, Flaxman SR, Price H, Jonas JB, Keeffe J, Leasher J, Naidoo K, Pesudovs K, Resnikoff S, Taylor H, Bourne RRA (2013). Global prevalence of vision impairment and blindness: magnitude and temporal trends, 1990–2010. Ophthalmology.

[CR44] Temkin-Greener H, Bajorska A, Peterson DR, Kunitz SJ, Diane G, Williams TF, Mukamel DB (2004). Social support and risk-adjusted mortality in a frail older population. Med Care.

[CR45] Terzian AS, Holman S, Nathwani N, Robison E, Weber K, Young M, Greenblatt RM, Gange SJ (2009). Factors associated with preclinical disability and frailty among HIV-infected and HIV-uninfected women in the era of cART. Journal of Women's Health.

[CR46] The United Republic of Tanzania (2017). National Guidelines for the management of HIV and AIDS. Ministry of Health Community Development Gender Elderly and Children, (ed): Dar es Salaam, pp 177.

[CR47] The World Bank (2019 ). Data for Tanzania

[CR48] Triant VA, Lee H, Hadigan C, Grinspoon SK (2007). Increased acute myocardial infarction rates and cardiovascular risk factors among patients with human immunodeficiency virus disease. J Clin Endocrinol Metab.

[CR49] UNAIDS (2017). United Republic of Tanzania In: Country factsheets.

[CR50] Van Damme W, Kober K, Kegels G (2008). Scaling-up antiretroviral treatment in Southern African countries with human resource shortage: how will health systems adapt?. Soc Sci Med.

[CR51] Webb Z (2018). The association of nutritional factors with cognitive impairment in ART-treated HIV-positive adults aged 50 and older in Northern Tanzania

[CR52] Willig AL, Overton ET, Saag MS (2016). The Silent Epidemic - Frailty and Aging with HIV. Total patient care in HIV & HCV.

[CR53] Yarnall AJ, Sayer AA, Clegg A, Rockwood K, Parker S, Hindle JV (2017). New horizons in multimorbidity in older adults. Age Ageing.

[CR54] Zhang Q, Guo H, Gu H, Zhao X (2018). Gender-associated factors for frailty and their impact on hospitalization and mortality among community-dwelling older adults: a cross-sectional population-based study. PeerJ.

